# The Effect of Melatonin on Periodontitis

**DOI:** 10.3390/ijms22052390

**Published:** 2021-02-27

**Authors:** Barbora Konečná, Paulína Chobodová, Jakub Janko, Lenka Baňasová, Janka Bábíčková, Peter Celec, Ľubomíra Tóthová

**Affiliations:** 1 Institute of Molecular Biomedicine, Faculty of Medicine, Comenius University, 811 08 Bratislava, Slovakia; basa.konecna@gmail.com (B.K.); paulina.chobodova@gmail.com (P.C.); jakubjanko7@gmail.com (J.J.); jana.babickova@gmail.com (J.B.); petercelec@gmial.com (P.C.); 2 Department of Stomatology and Maxillofacial Surgery, Comenius University, 812 50 Bratislava, Slovakia; lenkabanasova@hotmail.com; 3 Department of Clinical Medicine, University of Bergen, 5021 Bergen, Norway; 4 Department of Molecular Biology, Faculty of Natural Sciences, Comenius University, 841 04 Bratislava, Slovakia; 5 Institute of Pathophysiology, Faculty of Medicine, Comenius University, 811 08 Bratislava, Slovakia

**Keywords:** periodontal disease, antioxidant treatment, micro-computed tomography

## Abstract

Background: Periodontitis is a chronic disease with a complex etiology that includes bacterial colonization, excessive inflammation, and oxidative stress. The hormone melatonin has antioxidant properties and might contribute to alleviating chronic conditions by reducing oxidative stress. The aim of this study was to analyze the effect of exogenous melatonin on periodontitis in an animal model of the disease as well as in patients with periodontitis. Methods: In rats with ligature-induced periodontitis, melatonin was administered in drinking water for two weeks. In the human study, patients with treatment-resistant periodontitis were asked to rinse their mouths with a solution containing melatonin or placebo every evening for two weeks. Periodontal status as well as salivary markers of oxidative stress were assessed at the end of the study. Results: Neither radiography nor μCT revealed any significant effects of melatonin on alveolar bone loss. Gum recession was the only improved macroscopic measure in rats (*p* < 0.05). Analysis of salivary markers of oxidative stress revealed no effects of treatment in rats or humans despite clearly elevated melatonin concentrations in melatonin treated groups. Conclusion: Our results do not support the use of melatonin for the treatment of periodontitis. However, the negative outcome is limited by the short duration of the study and the chosen route of application as well as the dose of melatonin.

## 1. Introduction

Periodontitis is a set of chronic inflammatory diseases characterized by increased pathogens in the oral cavity, and increased activity of the host immune response. Yet, the complete etiology of the condition remains unknown [1]. Periodontitis develops from untreated gum infection (gingivitis), which may lead to the damage of tissue supporting the teeth, and loss of teeth. Periodontitis often worsens other health conditions, such as diabetes mellitus or other metabolic problems [2]. 

The normal oral microflora consists of commensal bacteria and pathogens in a dynamic balance. Dysbiosis in periodontal biofilms is included in the pathogenesis of periodontitis. This includes enhanced immune response and the augmentation of inflammatory response through local or systemic inflammatory mediators such as Il-6, Il-1 or CRP [3]. Host-derived cytokines released during microbial load have significant effects on the immune and inflammatory responses in periodontal disease [4]. Additionally, a shift in the composition of the microflora might result in worsened oral health. In periodontitis, pathogenic bacteria prevail. The metabolites of such bacteria irritate the mucosa, resulting in inflammation. Moreover, these metabolites affect the surface of teeth enamel, which can further impair the structural integrity of the teeth supporting tissue [5]. 

Due to the increased pathogens, a high number of neutrophils migrate towards the inflammation site. This results in proteases production and formation of reactive oxygen species. Thus, a high number of neutrophils fighting the pathogens leads to increased oxidative stress [6,7]. Oxidative stress leads to cell and tissue damage by various mechanisms. These include DNA damage, lipid peroxidation, and protein or enzyme oxidation [8,9]. There is a direct correlation between chronic periodontitis and oxidative stress in human saliva [10]. Several animal experiments showed that the severity of periodontitis was improved by the suppression of oxidative stress [6,11].

Several compounds with antioxidant actions proved beneficial in managing periodontitis [12]. Melatonin has helped to regulate immune response and prevent damage of periodontal tissue [13,14,15,16]. Similarly, vitamin C showed positive effect in treatment of periodontitis [17]. However, due to its acidity, vitamin C could contribute to teeth enamel damage in long-term run. Therefore, melatonin with its neutral pH could be considered to be a less harmful and superior option. Additionally, several recent studies showed that melatonin effect might go beyond antioxidant action. Locally administered melatonin increased the formation of trabecular bone near implant contact in rabbits [18]. Similarly, in rats, treatment by melatonin dissolved in drinking water increased bone volume and trabecular and cortical thickness of femurs, and had a direct stimulatory effect of melatonin on osteoblast differentiation and proliferation [19]. This effect was not described in vitamin C. Melatonin is secreted by the pineal gland in circadian cycles, mainly during sleep [16]. However, a small portion is also produced by the salivary glands [20]. Therefore, we chose melatonin to test if it might contribute to the prevention of severe oral inflammation and improve the bone detachment to inflamed gingival tissue. 

The aim of our work was to analyze the local effects of melatonin in experimental periodontitis and in patients with refractory periodontitis, with a specific focus on saliva as a diagnostic fluid.

## 2. Material and Methods

### 2.1. Animals 

Forty Wistar male rats (16–19 weeks old) were purchased from Anlab (Prague, Czech Republic). Animals were housed in an animal facility with controlled temperature (22–24 °C), humidity (45–55%), and 12/12 h light/dark cycle. Animals had access to water and standard chow pellets ad libitum. 

The rats were randomly divided into following groups: CTRL group—rats without ligature and drinking water, CTRL+MEL group—rats without ligature and drinking water with melatonin, LIG group—rats with ligature and drinking water, and LIG + MEL group—rats with ligature and drinking water with melatonin.

### 2.2. Induction of Periodontitis 

The periodontitis in the rats of experimental groups LIG and LIG+MEL was induced by three-fold ligature of the first right lower molar in the neck area of the tooth. The procedure was performed under general anesthesia induced by intraperitoneal ketamine/xylazine administration, 100/10 mg/kg, respectively. The non-absorbable thread 7-0 was used and left at place for 4 weeks to induce periodontitis [15]. Standard food pellets were replaced by soft biscuits to ensure the stability of the ligature. The presence of the ligature was controlled every two weeks. The presence of ligature around the tooth prevents natural cleaning and allows bacteria to spread excessively. After 4 weeks, the water of CTRL+MEL and LIG+MEL rats was replaced by melatonin solution (Sigma Aldrich, St. Louis, MO, USA, from 7:00 am to 07:00 pm) for two weeks calculated according to water intake and corresponding to ~10 mg/kg. Animals were sacrificed under general anesthesia by ketamine/xylazine (100/10 mg/kg). The degree of inflammation of affected area was determined according to macroscopic score (0-3; without change = 0, swelling = 1, redness = 2, gradual exposure of the tooth root = 3) by a blinded examiner. The exposure of tooth root, and changes on the alveolar bone, were analyzed using micro-CT and X-ray pictures (Spectrum CT In Vivo Imaging System (IVIS), PerkinElmer, Waltham, MA, USA). Saliva samples were collected under general anesthesia induced by the intraperitoneal application of pilocarpine (1 mg/kg) at the end of the experiment.

### 2.3. Real-Time PCR Analysis 

To quantify the presence of bacterial colonization in rats, two hundred microliters of saliva were used for bacterial DNA isolation using commercial membrane-based kit QIAamp DNA Kit (Qiagen, Hilden, Germany) according to the instructions of the manufacturer. DNA was eluted in 30 µL of the RNase free water. Afterwards, the bacterial DNA was quantified using the 16SrRNA qPCR assay (QuantiFast SYBR Green PCR Master Mix, Qiagen, Hilden, Germany) (F: AGACTCCTACGGGAGGCAGCAGTT and R: GWATTACCGCGGCKGCTGGCAC) on the MasterCycler RealPlex (Eppendorf, Hamburg, Germany). The amount of bacterial DNA is presented as genome equivalents per ml of saliva. Standard curve was generated using Escherichia coli 25922 (American Type Culture Col- lection, Manassas, VA, USA). 

### 2.4. Clinical Study

This study was a blinded randomized and placebo-controlled pilot study that was approved by the Ethics Committee of the Institute of Pathophysiology, Medical Faculty, Comenius University in Bratislava. All patients were treated in concordance with Declaration of Helsinki. 

Twenty age- and gender-matched patients with refractory periodontitis were randomly assigned into placebo (PLACEBO, *n* = 10; age: 47 ± 6) or melatonin (MEL, *n* = 10, age: 44 ± 7) group. The periodontitis was diagnosed based on the current guidelines [21] with inclusion criteria as follows: A) presence of at least 20 teeth, B) at least 40% sites with a probing depth (PD) ≥ 4mm and clinical attachment level (CAL) ≥ 2mm, C) more than 40% of sites with bleeding on probing (BOP), and D) at least two sites with radiographically alveolar bone loss (ABL) of ≥ 2 mm verified by radiography. The periodontitis was further evaluated by a single-skilled examiner where plaque index (PI), sulcus bleeding index (SBI), and bleeding on probing (BOP) were determined [22,23,24,25]. Additionally, patients with periodontitis on treatment for at least one year with no evident improvement were included in the study, which is consistent with refractory periodontitis.

### 2.5. Sampling

**Figure 1:**
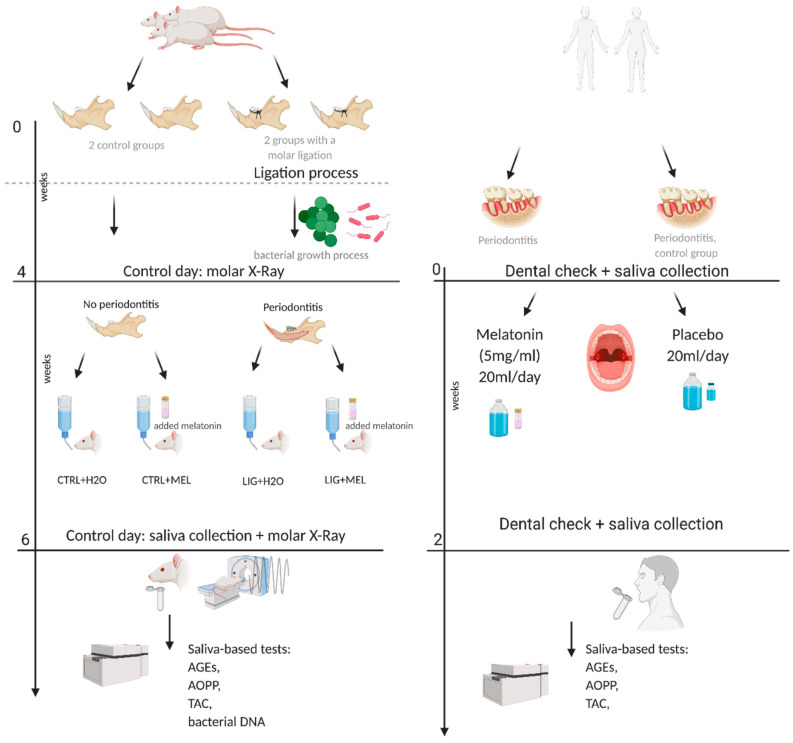
Graphical design of the experimental and clinical study. Created with BioRender.com.

Patients were examined, and their saliva was sampled at a single Dental Clinic in Bratislava, Slovakia. Melatonin (Sigma Aldrich, St. Louis, MO, USA) was administered to all patients in MEL group as 20 mL melatonin solution (5 mg/mL), which was used to rinse the oral cavity before the sleep and after brushing the teeth for 14 days. Five milliliters of saliva were collected before and 2 weeks after the treatment. After the saliva collection, the clinical examination was performed as described above. The same procedure was carried out with all PLACEBO patients, with vehicle without melatonin. All participants were asked not to eat or drink and to perform oral hygiene 1 h prior to sampling. The inclusion criteria included age above 18 years and otherwise healthy with no evident acute illness. All participants signed informed consents. The design of the clinical and experimental study is illustrated in Figure 1. 

The exclusion criteria were defined as age below 18 years; smoking and former smoking patients; patients taking supply of per oral vitamins on a regular basis; and the presence of chronic systemic diseases including but not limited to hypertension, chronic heart disease, alcohol abuse, cirrhosis, chronic kidney disease, and leukoplakia. The patients with signs and symptoms of acute illness or pregnant females were also excluded. Lastly, patients on antibiotic treatment for the last three months, and patients using non-steroid anti-inflammatory drugs for the last 6 weeks and/or immunosuppressive medications using for last 6 months, were not included in the study. 

### 2.6. Melatonin Concentration Measurement 

Concentrations of melatonin in saliva were analyzed using melatonin ELISA (Salimetrics, Carlsbad, CA, USA), following the instructions of the manufacturer. The concentrations of melatonin were calculated from calibration curve of the standards solutions. 

### 2.7. Oxidative Stress

Markers of oxidative stress analyzed in the samples included advanced glycation end products (AGEs) and advanced oxidation protein products (AOPP). Measures of antioxidant status assessed in this study were total antioxidant capacity (TAC), as described in detail before [26,27].

### 2.8. Statistical Analysis 

All statistical analyses were performed by GraphPad Prism version 8.0 (La Jolla, CA, USA). The differences in oxidative stress markers between the beginning and end of the clinical study were analyzed using non-parametric Wilcoxon matched pairs signed rank test. As for the differences between the CTRL and MEL groups, the data were analyzed using non-parametric Mann–Whitney test. 

The differences among the groups in animal study were assessed using one-way ANOVA with the subsequent Bonferroni post-hoc correction. In both studies, values *p* < 0.05 were taken as statistically significant. The data are presented as the mean ± SD. 

## 3. Results

### 3.1. Morphometric Changes in Rats

**Figure 2:**
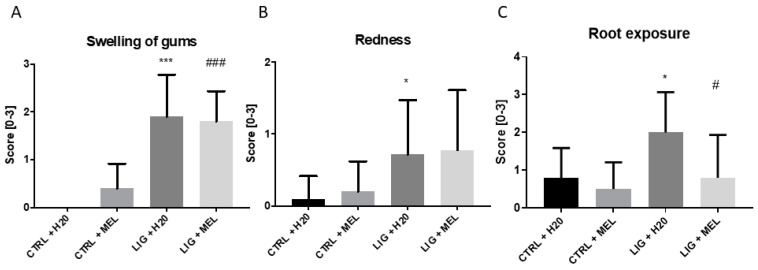
Macroscopic score of swelling (**A**), redness (**B**), and root exposure (**C**) in individual groups. * denotes *p* < 0.05 vs. CTRL + H2O; *** denotes *p* < 0.001 vs. CTRL + H2O; # denotes *p* < 0.05 vs. LIG + H2O; ### denotes *p* < 0.001 vs. LIG + H2O.

**Figure 3:**
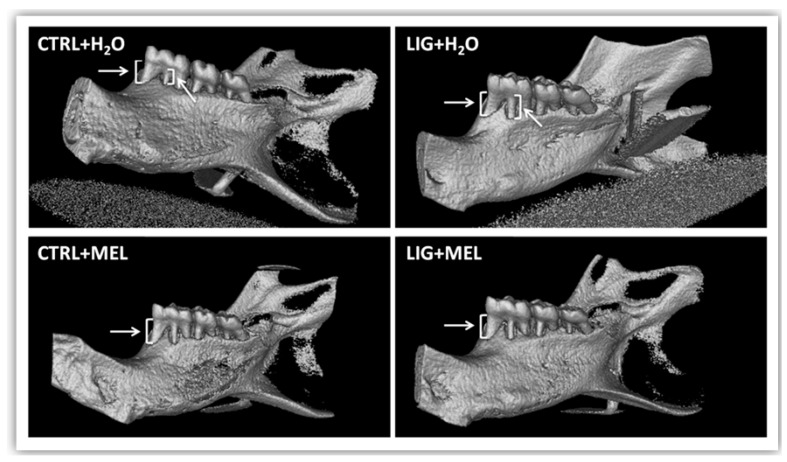
µCT pictures of molars after removal of ligature. (**A**) CTRL + H2O. (**B**) LIG + H2O. (**C**) CTRL + MEL. (**D**) LIG + MEL.

Macroscopic evaluation in rats showed a larger degree of gums swelling in animals with ligature when compared to control group (*p* ˂ 0.001; *t* = 6.9; Figure 2A). Similarly, redness between LIG + H2O group and CTRL + H2O group was significantly higher in LIG + H2O group (*p* < 0.05; *t* = 2.3; Figure 2B). No significant difference was found between groups LIG + H2O and LIG + MEL in redness (*p* = n.s.; Figure 2B). Melatonin reduced the root exposure in LIG + MEL rats in comparison to LIG + H2O rats (*p* ˂ 0.05; *t* = 2; Figure 2C). The difference was found also between CTRL + H2O and LIG + H2O group (*p* ˂ 0.05; *t* = 2.7; Figure 2C). Micro CT pictures confirmed the larger root exposure in the group of animals with ligature (Figure 3B) in comparison to the control group (Figure 3A). Melatonin treatment reduced the exposure of tooth root in LIG + MEL (Figure 3D) in comparison with LIG + H2O group (Figure 3B). 

### 3.2. Bacterial DNA

**Figure 4:**
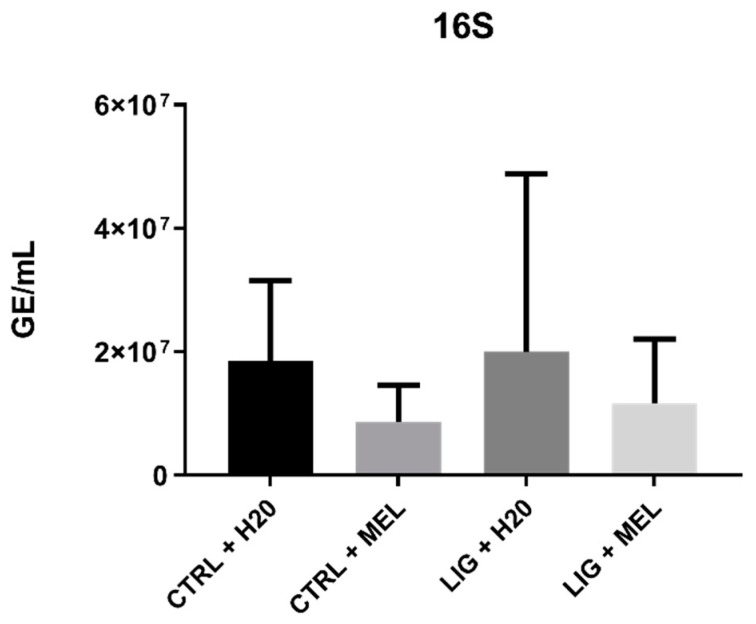
The genome equivalents of bacterial DNA in saliva.

There was no significant difference in concentration of bacterial DNA in saliva among the groups (*p* = n.s., Figure 4); however, there was numerically lower bacterial DNA in groups with melatonin (*p* = n.s., Figure 4). 

### 3.3. Melatonin Concentration and Oxidative status in Rats

**Figure 5:**
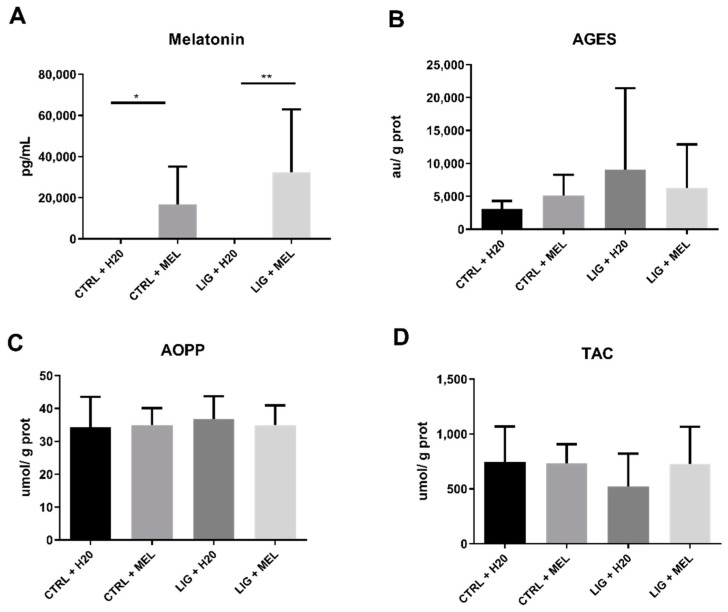
Salivary concentrations of melatonin (**A**) and oxidative status markers (**B**–**D**) in animal model of periodontitis. * denotes *p* < 0.05, ** denotes *p* < 0.01.

Melatonin concentration was significantly higher in LIG + MEL group compared to LIG + H2O group (*p* < 0.01; *t* = 3.2; Figure 5A) and in CTRL + MEL group compared to CTRL + H2O group (*p* < 0.05; *t* = 2.7; Figure 5A). There was no significant difference in AGEs, AOPP, and TAC between CTRL and LIG groups, but there were numerically higher concentrations of AOPP and numerically lower concentrations of TAC in CTRL + H2O group (*p* = n.s.; Figure 5B–D). 

### 3.4. Clinical Data of Patients

There were no significant differences in BOP, SBI, and PI scores before and two weeks after the treatment with melatonin. Data are shown in Table 1.

### 3.5. Melatonin Concentrations and Salivary Markers of Oxidative Status in Patients

**Figure 6:**
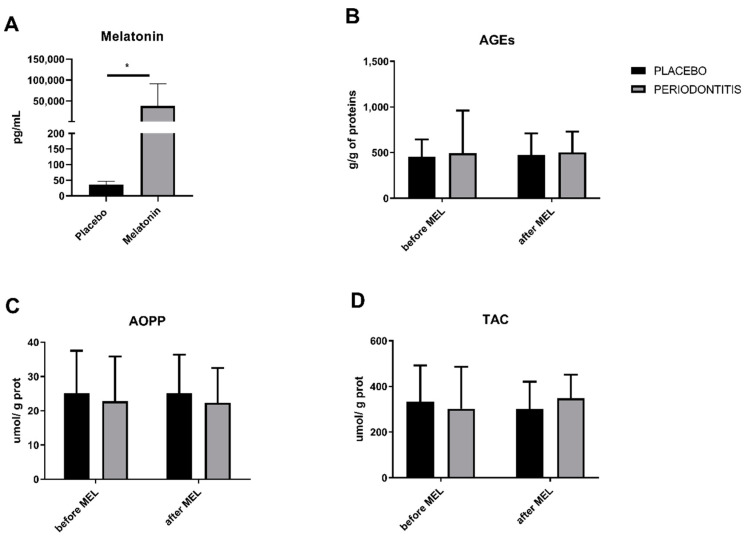
Salivary concentrations of melatonin (**A**) and oxidative status markers (**B**–**D**) in patients with periodontitis before and after 14 days of melatonin treatment. * denotes *p* < 0.05.

Melatonin was significantly higher in patients supplied with melatonin compared to placebo-supplied patients (*p* < 0.05; *t* = 2.3; Figure 6A). Markers of oxidative stress, AGEs, AOPP, as well as TAC did not to differ between placebo group and melatonin group (*p* = n.s.; Figure 6B–D). 

## 4. Discussion

In the current study, we performed a short-termed animal experiment and a clinical study in a similar setting. Our results in animal experiment showed a reduction in clinical markers such as redness, root exposure, and swelling after two weeks of melatonin treatment, but no changes in salivary markers of oxidative stress. In the small clinical trial, despite an increased melatonin in saliva, no significant changes either in clinical markers or salivary markers were found. 

Melatonin is one of the natural antioxidants produced by the pineal gland during dark phase of day, and in small amounts it is also stored and released by salivary glands [28]. The exogenous supply of melatonin could have antioxidant, anti-inflammatory, and anti-apoptotic effects [29]. Periodontitis is a chronic inflammatory disease. Its clinical symptoms include swelling, reddening, and formation of periodontal pockets [1]. As in other chronic inflammatory diseases, the dysbalance between antioxidants and reactive oxygen species causing oxidative stress occurs during periodontitis [8,30]. In our study, despite a high macroscopic score and inflammation in rats, oxidative stress markers in saliva did not differ significantly. However, markers of oxidative stress, especially AGEs, were numerically highest in ligated placebo treated group. Similarly, TAC seemed to decline in the placebo group in comparison to melatonin-treated groups. Additionally, bacterial DNA in saliva points towards a higher trend in LIG + H2O group in comparison to LIG + MEL group. Whether these results reflect the limitations of pilocarpine-induced salivation, and low levels of these markers in such saliva, requires further research in this area. Accordingly, similar studies in rats with periodontitis showed lower oxidative stress and inflammation in gingival tissue after melatonin treatment [15,31]. In our study, the importance was put on saliva as a diagnostic material. Additionally, bacterial DNA increases during periodontitis, reflecting increased bacterial load in oral cavity and in saliva in humans [32,33]. Similar to oxidative stress markers, pilocarpine-induced saliva might not reflect the actual levels of bacterial DNA. 

In humans, saliva represents an interesting bodily fluid for non-invasive sample collection and diagnostics, but this approach is not yet widely used in clinical practice. In our clinical study, we included patients that did not show any improvement in periodontitis after one year of treatment. According to the results, scoring parameters as well as oxidative stress markers did not show any improvement after the melatonin treatment. While the dose could be considered as a high dose, the length of the melatonin treatment was chosen to match the animal model [34], which proved as insufficient. This is probably the main limitation of the presented study. Whether a prolonged treatment or even higher dose of melatonin would have a beneficial effect in periodontitis is currently unknown and should be further studied. However, melatonin treatment in diabetic patients with periodontitis reduced inflammation and bacterial load [35]. Compared to the present study, melatonin was administered orally, suggesting that systemic effects might have a longer lasting effect, compared to the present, local treatment by mouth washing. Nevertheless, the local treatment with melatonin might be an alternative approach reducing possible unwanted systemic effects. Additionally, the measurement of the salivary antibodies IgG and IgA could provide some insight into the stage of periodontitis. In our study, the periodontitis was severe refractory with no effect on salivary oxidative stress markers within the two-week period of melatonin treatment [36], but milder forms of periodontitis could respond faster. 

Local application of vitamin C decreased the oxidative stress in a rodent model of periodontitis [37]. A combination of melatonin with appropriate doses of vitamin C could have a positive effect as a periodontitis treatment while not affecting teeth enamel due to the acidity. However, the human patients were resistant to standard treatment in our study, and to our knowledge, there is no animal model for such a condition. The biochemical markers of oxidative stress did not show any significant difference before and after melatonin treatment, although the antioxidant and anti-inflammatory effect of melatonin has been previously recognized [38]. However, these markers are universal and non-specific. Several recent studies point towards utilization of new markers such as soluble urokinase-type plasminogen activator receptor (suPAR) for monitoring of periodontitis severity. SuPAR is a mediator present in various body fluids including saliva [39]. Additionally, during periodontitis it is highly expressed in macrophages, monocytes, and T-lymphocytes, i.e., during inflammation suPAR might be a valuable prognostic factor for periodontitis [3] and might help to categorize patients benefiting from a treatment.

Periodontitis does not belong to life-threatening diseases; however, its high incidence, protracted treatment, and reduced quality of life in long-term run make it a debilitating disease. Future studies should focus on the administration routes and effective time duration of melatonin treatment as well as on the utilization of saliva as a diagnostic body fluid for periodontitis in different stages of the disease. 

## Figures and Tables

**Table 1:** Clinical parameters of patients with periodontitis before and after the treatment with melatonin.

Parameters	Placebo (Before Treatment)	Placebo (After Treatment)	Melatonin (Before Treatment)	Melatonin (After Treatment)
**AGE** **[years]**	47 ± 6		44 ± 7	
**BOP**	65.69 ± 11.99	60.73 ± 11.50	60.15 ± 20.99	52.68 ± 21.53
**SBI**	3.06 ± 0.52	2.821 ± 0.41	2.581 ± 0.87	2.37 ± 0.88
**PI**	1.53 ± 0.94	1.38 ± 0.91	1.69 ± 1.34	1.45 ± 1.34

Data are expressed as mean ± SD. BOP—bleeding on probing; SBI—sulcus bleeding index; PI—plaque index. BOP: (yes/no; sum of “yes” spots x 100), SBI: (1—healthy, 2—slight bleeding on probing, 3—bleeding in probing, 4—edema and bleeding on probing, and 5—spontaneous bleeding); PI: (1—not present, 2—slight, and 3—medium). No significant changes were observed in clinical markers before and after treatment.

## Data Availability

Data generated and analyzed in this study are available from the authors upon request.
